# Vivid Dreams and Nightmares as an Adverse Effect of Beta‐Blockers in the Treatment of Episodic Migraine

**DOI:** 10.1155/crnm/5581364

**Published:** 2026-01-05

**Authors:** Carlos R. Silva-Rosas, Heather Angus-Leppan, Alonso H. Quijada, Andrés G. Briceño

**Affiliations:** ^1^ Department of Neurology & Neurosurgery, Clinical Hospital of University of Chile, Santiago de Chile, Chile; ^2^ Royal Free London NHS Foundation Trust, Pond Street, London, NW3 2QG, UK, nhs.uk; ^3^ Department of Clinical and Movement Neurosciences, UCL Queen Square Institute of Neurology, London, UK

**Keywords:** beta-blockers, metoprolol, migraine prophylaxis, neuropsychiatric adverse effects, nightmares, propranolol

## Abstract

Beta‐adrenergic blockers are effective in migraine prevention but can induce neuropsychiatric side effects, including vivid dreams and nightmares. Their lipophilicity allows penetration of the central nervous system, where β1‐adrenergic blockade may disrupt REM sleep, alter noradrenergic activity, and suppress melatonin secretion, contributing to emotionally intense dreams. While reported in cardiovascular patients, this adverse effect remains underrecognized in migraine therapy. Adult patients were identified from those seen in the outpatient department of the Clinical Hospital of University of Chile in Santiago between 2022 and 2024. We present three cases of patients with episodic migraine with aura who developed distressing, recurrent nightmares after initiating propranolol, or metoprolol. Symptoms emerged shortly after treatment initiation and resolved upon discontinuation. Nightmare content involved emotionally distressing themes, leading to significant psychological discomfort. Clinicians should be aware of vivid nightmares as a potential adverse effect of lipophilic beta‐blockers in migraine prevention. Understanding this may enable patients to tolerate the symptom. If it impacts adherence and/or quality of life, a treatment change will be needed.

## 1. Introduction

The optimal selection of preventive treatment for migraine is individualized, requiring multiple considerations in deciding on specific medications and nonpharmacologic approaches [1–6]. Currently, calcitonin gene‐related peptide (CGRP)–targeting therapies are recommended as a first‐line approach for migraine prevention, alongside traditional first‐line treatments, without the necessity of prior failure of other migraine preventive treatments [1, 2]. However, economic factors significantly influence clinical decision‐making in migraine management [1–3].

Traditional preventive migraine treatments were initially developed for other medical conditions before being adopted for migraine [1–5]. While clinical evidence supports their efficacy, these treatments are associated with concerns regarding tolerability, safety, and the absence of clear predictors for treatment response [1]. Nonspecific migraine treatments continue to be widely used in many countries [4–6].

Beta‐adrenergic blocking drugs are an established treatment option with proven efficacy for episodic migraine, with and without aura [1–6]. These medications are long‐standing, widely available, and cost‐effective [3, 6]. Their exact mechanisms of action in migraine prophylaxis remain unclear [4], and they are associated with neuropsychiatric adverse effects, including depression, fatigue, insomnia, drowsiness, hallucinations, and unusual dreams or nightmares [7–10]. There are a few reports linking beta‐blockers to neuropsychiatric symptoms (particularly depression and sleep disturbance) in elderly patients with cardiovascular disease, and these patients may have an increased susceptibility [8, 9]. There are no reports of their association with migraine treatment. Consequently, those adverse effects may go unnoticed by clinicians and remain underdiagnosed or incorrectly attributed to other causes.

We present three cases and review the potential mechanisms underlying these unique adverse effects to beta‐adrenergic blocking drugs.

## 2. Case Presentation

Adult patients were identified from those seen in the outpatient service of a secondary care teaching hospital in Santiago, Chile, between 2022 and 2024.

### 2.1. Case 1

A 22‐year‐old previously healthy Hispanic female had a history of migraine and visual auras since age 13, and her diagnosis of episodic migraine with aura was based on the International Classification of Headache Disorders, third edition (ICHD‐3) [11]. She was taking oral contraceptives. The patient experienced four monthly headache days (MHDs) [12] and had a Migraine Disability Assessment (MIDAS) [12] score of 12, indicating moderate disability. She had never received preventive treatment. Neurological examination and brain MRI were normal. She was prescribed propranolol 40 mg twice daily, along with naratriptan 2.5 mg for acute attacks.

One week after initiating propranolol, she complained of vivid nightmares every night, featuring distressing scenarios such as the death of relatives and being chased by wild animals. These episodes caused significant anxiety, and she persistently recalled the nightmares throughout the day, leading to emotional distress. Propranolol was discontinued, and topiramate 25 mg once daily was introduced. The nightmares resolved immediately.

### 2.2. Case 2

A 32‐year‐old previously healthy White female with a history of migraine without aura since age 20, diagnosed as per ICHD‐3 [11] criteria. She was using oral contraceptives and reported five MHDs, with a MIDAS [12] score indicating severe disability. Neurological examination and brain MRI were unremarkable. She had previously been treated with topiramate 50 mg daily and eletriptan 40 mg for attacks. Topiramate was effective but was discontinued due to drowsiness and memory impairment.

Two weeks after starting propranolol 60 mg twice daily, she developed recurrent vivid nightmares in which she found herself alone in a cemetery with open graves. The distressing nightmares persisted, leading to significant psychological discomfort. Propranolol was discontinued, and flunarizine 5 mg once daily was initiated. The nightmares resolved completely.

### 2.3. Case 3

A 25‐year‐old previously healthy Hispanic male with a history of migraine with aura since age 18, diagnosed as per ICHD‐3 [11] criteria. He experienced four MHDs and had a MIDAS [12] score of 14, indicating moderate disability. He had been treated with divalproex sodium 500 mg once daily and eletriptan 40 mg for attacks. While divalproex sodium was effective, it was discontinued due to weight gain and the development of metabolic syndrome. Neurological examination and brain MRI were normal.

He was then prescribed metoprolol 50 mg daily. After 2 weeks, he experienced intermittent nightmares, which became more frequent, occurring nightly. His nightmares revolved around workplace failures, termination from his job, and relationship conflicts involving infidelity. Metoprolol was discontinued, and topiramate 100 mg once daily was initiated. The nightmares ceased.

Table 1 provides an overview of the clinical cases presented.

**Table 1:** Summary of the clinical cases.

Case	ICHD‐3^a^	MHDs^b^	MIDAS^c^ score	Beta‐blocker	Nightmares
25‐year‐old Hispanic female	Episodic migraine without aura	4	12 (moderate)	Propranolol 80 mg/day	Vivid nightmares every night: “Death of her relatives and being chased by wild beasts”
32‐year‐old White female	Episodic migraine with aura	5	22 (severe)	Propranolol 120 mg/day	Recurrent vivid nightmares: “She was alone in a cemetery and the graves were open”
25‐year‐old Hispanic man	Episodic migraine with aura	4	14 (moderate)	Metoprolol 50 mg/day	Recurrent vivid nightmares: “Poor performance on his job and he is fired. Infidelity of his partner”

^a^ICHD‐3: International Classification of Headache Disorders, third edition.

^b^MHDs: monthly headache days.

^c^MIDAS: Migraine Disability Assessment. Score 5–10: mild disability, 11–20: moderate disability, and 21+: severe disability.

## 3. Discussion

### 3.1. Nightmares and Migraine

Sleep disorders and pain, including migraine, are frequently comorbid, and the relationship is bidirectional [13]. Nightmare disorders are distinguished from intermittent nightmares by their associated distress and impact, which may significantly reduce quality of life and exacerbate or cause mood disorders [13]. This report highlights the link between beta‐blockers treatment in migraine and nightmare disorders. Symptoms were sufficiently distressing to warrant treatment cessation, even when successful in headache reduction. Nightmares are reported in association with beta‐blocker use in cardiovascular diseases, but this is the first case series in migraine. Thus, the effect is not condition‐specific. Further studies are needed to investigate the frequency of nightmare disorders in migraine and potential links with other treatments.

### 3.2. Pharmacokinetics of Beta‐Blockers

The ability of beta‐blockers to cross the blood–brain barrier is determined by their lipid solubility (octanol/water partition coefficient or log *P*) and protein‐binding properties. Propranolol, metoprolol, and pindolol exhibit high lipid solubility, enabling them to penetrate the central nervous system (CNS) efficiently [10, 14]. This characteristic is critical for their efficacy in migraine prevention, with evidence supporting their use at Level A1 [1–6].

Conversely, hydrophilic beta‐blockers, such as atenolol and nadolol, have limited CNS penetration. Brain concentrations of lipophilic agents such as propranolol and metoprolol are 10–20 times higher than those of atenolol; for instance, the brain‐to‐plasma ratio for propranolol is 26:1, whereas for atenolol, it is 0.2:1 [9]. Thus, atenolol and nadolol have less efficacy as preventive treatment of migraine [1–5].

### 3.3. Pathophysiology of Beta‐Blocker–Induced Vivid Nightmares

Beta‐blockers exert CNS effects by blocking β‐adrenergic receptors, particularly in the amygdala and prefrontal cortex, which are key regions involved in stress regulation, emotional memory, and sleep modulation [15]. Propranolol and metoprolol decrease central sympathetic activity via β1–receptor blockade, potentially disrupting the regulation of rapid eye movement (REM) sleep [16–18].

Reduced noradrenergic activity may trigger compensatory mechanisms that heighten REM sleep intensity, leading to more vivid and emotionally charged dreams. Beta‐blockers have been reported to prolong REM latency and alter REM sleep distribution, which may increase the likelihood of distressing nightmares [17, 18]. Additionally, beta‐blockers suppress melatonin secretion in susceptible individuals [17], further contributing to sleep disturbances. Dopamine receptor stimulation may represent another common mechanism underlying beta‐blocker–induced nightmares [15, 18]. The β1‐selectivity of metoprolol could explain its comparatively lower frequency of this adverse effect relative to propranolol [14].

**Figure 1:**
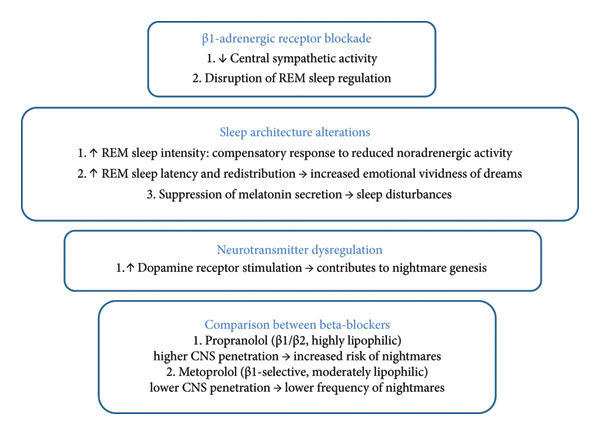
Pathophysiology of beta‐blocker–induced vivid nightmares demonstrating roles of receptor blockade, sleep architecture, neurotransmitters, and lipophilia [15–18]. REM: rapid eye movement; CNS: central nervous system.

Figure 1 provides a summary of the relationship between beta‐blockers and sleep.

## 4. Causality

The beta‐blockers fulfilled probable/likely causality for nightmares using the WHO–UMC criteria and probable causality on the more stringent Naranjo scale (the reaction followed a reasonable temporal sequence after a drug, followed a recognized response to the suspected drug, was confirmed by withdrawal but not by exposure to the drug, and could not be reasonably explained by the known characteristics of the patient’s clinical state) [19].

Most importantly, there was no other explanation that followed the appearance and resolution of the nightmares.

## 5. Limitations

Standardized tools were not used to assess sleep disturbance or vivid dreams, and this could have reduced ascertainment. Nightmares are difficult to assess as available strategies are psychometric, self‐reported, and subjective [20]. Formalized prospective questionnaires are susceptible to expectancy bias and overestimation, while retrospective ones may underestimate nightmare frequency because of recall bias. A strong argument for the methodology used is that informal assessment reflects the real‐world practice of most clinicians.

Patients were not formally assessed with psychiatric evaluation. Depression and anxiety were excluded clinically by the treating neurologists (CSR, AHQ, and AGB). The rapid resolution of symptoms argues strongly that the beta‐blockers were causal.

CGRP‐targeting therapies are recommended by some as a first‐line approach for migraine prevention [1]. However, among traditional treatments, beta‐blockers remain an effective and cost‐efficient option [3, 6]. Despite their efficacy, beta‐blockers can be associated with significant adverse effects. Lipophilic beta‐blockers, such as propranolol and metoprolol, have been linked to neuropsychiatric adverse effects, including vivid dreams and nightmares. Although this adverse effect has been sporadically reported in the literature, primarily in the context of cardiovascular pathology and older adults, no cases have been reported in migraine. It appears to be underrecognized by clinicians and is likely underdiagnosed. Incorrect attribution of nightmare disorders may reduce quality of life in migraineurs and result in treatment of an iatrogenic complication. In some patients with improvement on beta‐blockers but mild and tolerable vivid dreams, an understanding of the cause may be enough. In others, as in the cases presented here, nightmares may be an adverse effect requiring a switch in treatment.

**Nomenclature**
CGRP: Calcitonin gene‐related peptide
CNS: Central nervous system
ICHD‐3: International Classification of Headache Disorders, 3rd edition
MHDs: Monthly headache days
MIDAS: Migraine Disability Assessment
MRI: Magnetic resonance imaging
REM: Rapid eye movement

## Consent

All patients gave informed consent to their inclusion in anonymized form in the publication.

Case records were anonymized, and no identifiable data were stored, in accordance with Law No. 21.719 (New Data Protection Law, 2024) of Chile and General Data Protection Regulation, European Union, 2016.

## Disclosure

The authors have nothing to report.

## Conflicts of Interest

The authors declare no conflicts of interest.

## Author Contributions

Carlos R. Silva‐Rosas: conceptualization, methodology, investigation, formal analysis, supervision, validation, visualization, software, project administration, writing–original draft, and writing–review and editing.

Heather Angus‐Leppan: formal analysis and writing–review and editing.

Alonso H. Quijada: data curation and writing–review and editing.

Andrés G. Briceño: data curation and writing–review and editing.

## Funding

No funding was received for this manuscript.

## Data Availability

Anonymized data are available for researchers to review on application to Professor Silva‐Rosas.
